# Pathways to Interleukin-6 in Healthy Males and Serious Leisure Male Athletes: Physical Activity, Body Composition and Age

**DOI:** 10.1371/journal.pone.0040513

**Published:** 2012-07-10

**Authors:** Leah FitzGerald, Paul M. Macey, Mary-Lynn Brecht

**Affiliations:** 1 School of Nursing, University of California Los Angeles, Los Angeles, California, United States of America; 2 School of Medicine, Semel Institute, Integrated Substance Abuse Programs, University of California Los Angeles, Los Angeles, California, United States of America; Oklahoma State University, United States of America

## Abstract

Physical activity (PA) is beneficial to overall health, in part due to physiological changes that lower risk factors for cardiovascular disease, including reduced inflammation. However, the mechanism by which PA reduces inflammation is unclear. One possible pathway is that PA improves body composition which in turn reduces inflammation. To test this hypothesis, we used structural equation modeling (SEM) to assess PA-body composition –inflammation pathways, as well as influences of age. In a sample of 72 healthy males with a range of PA profiles (age 18–65, mean ±sd  =  ), we measured PA as metabolic equivalent tasks (as per the International PA Questionnaire), body composition as percent body fat, lean mass, and fat mass, and inflammation as plasma interleukin-6 (IL-6). We treated body composition in the SEM analysis as a latent variable indicated by the three measures. We performed statistical corrections for missing values and one outlier. The model demonstrated significant effects of PA on IL-6 both directly and through body composition. Percent body fat, fat mass, and lean mass were significant indicators of the body composition latent variable. Additionally, age showed an indirect effect on IL-6 through body composition, but no direct effect. The findings suggest that PA does improve inflammatory profile through improving body composition, but that other pathways also exist.

## Introduction

Circulating Interleukin 6 (IL-6) levels are an independent predictor of cardiovascular mortality among otherwise healthy adults [1], and elevated plasma levels of this cytokine are associated with higher body mass index (BMI), percent body fat, and overall morbidity [2]. In addition to cardiovascular disease (CVD), IL-6 dysregulation is implicated in the pathology of several diseases including rheumatoid arthritis, osteoporosis, and various forms of cancer [3]. Furthermore, elevated plasma IL-6 levels are prospectively associated with an increased cardiovascular risk in initially healthy individuals [4]. Apparently healthy men in the highest quartile of baseline IL-6 concentration show more than a twofold higher risk of myocardial infarction than men in the lowest IL-6 quartile after a six year follow up [5]. Exercise intervention programs reduce systemic low-level inflammation in patients with CVD and in healthy controls, [6] suggesting regular exercise mediates suppression of the IL-6 inflammatory response. Long-term exercise training reduces plasma inflammatory states and higher levels of physical activity are associated with reduced levels of peripheral inflammatory mediators compared with more sedentary lifestyles [7]. However, the pathways by which IL-6 is reduced through exercise are not fully understood [8].

IL-6 is a member of a family of mediators involved in regulation of the acute-phase response to injury and infection and inflammation, and chronic elevations in IL-6 are thought to represent a state of atherosclerotic inflammation [9]. Physical activity correlates with IL-6 levels, but the relationship is complex. Acute exercise consistently increases circulating levels of IL-6 and the source of this increase is thought to be primarily from muscle tissue and to a much lesser extent adipose tissue, [10] however, with chronic exercise, markers of chronic inflammation decrease [11]. A considerable literature documents the effects of long-term exercise on health outcomes [7].

Acute moderate intensity [12] exercise and prolonged strenuous exercise [13] are associated with acute phase response, with prolonged effects decreasing after exercise, suggesting that cytokine inhibitors and anti-inflammatory cytokines may restrict the degree and extent of the inflammatory response to exercise. IL-6 mediates many aspects of exercise-induced acute-phase response [14] and following acute exercise, systemic levels of inflammatory response mediators such as IL-6 demonstrate significant increases; however, the precise change of the exercise-induced change remains speculative, [15] with differences observed by sex, [16] exercise intensity [17] and age [18]. It is unclear whether long-term vigorous physical activity acts directly or indirectly through other factors to reduce IL-6 levels. Body composition has an independent influence on inflammation, so physical activity may work in an indirect manner through body composition to reduce inflammation. Age is associated with changes both in inflammation and body composition, but it is unclear whether age affects inflammation directly or indirectly through body composition. For example, older adults (mean age 69±9 years) [19] show an indirect effect of age on IL-6 via body mass, which is one aspect of body composition. The same study found an indirect pathway from exercise through body mass, but no direct pathway.

The purpose of this cross-sectional study was to examine physical activity and body composition influences on circulating IL-6 levels. We studied healthy males with a wide range of physical activity profiles, including serious leisure male athletes cyclists (cycled >8 hours/week), male triathletes (trained >5 hours/week), male recreational athletes (exercised ≤30 minutes moderate intensity exercise most days), and sedentary males.

## Methods

### Objectives

We examined whether self-reported physical activity, body fat percentage, fat mass and lean mass were associated with IL-6 levels and whether the relationship between activity and IL-6 was accounted for by these variables. We hypothesized that IL-6 would be influenced by physical activity via body composition.

### Participants, Measurements and Protocol

Seventy-three medically healthy men (age 18–65 years) participated in the study, including a subset of recreational endurance male athletes (cyclists), to ensure a range of physical activity levels in the sample. (One subject was omitted from analyses because of distributional considerations; see analysis section for details.) Participants were free from co-morbid conditions affecting inflammatory markers and any known cardiovascular, respiratory or metabolic disorders. Participants did not take any medications, including over-the-counter pain/anti-inflammatory medication and were non-smokers. Participants gave written consent prior to the investigation. All research involving human participants for this study was specifically approved by the University of California, Los Angeles institutional review board for use of Human subjects according to the Declaration of Helsinki. Information collected regarding age and race/ethnicity was based on self-report. Participants had previously participated in a study examining bone health [20].

Physical activity was measured with the *International Physical Assessment Questionnaire* (IPAQ), a detailed questionnaire that quantifies activity as a number of metabolic equivalent tasks (METS) per week. IPAQ estimates weekly time spent in different dimensions of physical activity and of physical inactivity. Total physical activity level was calculated and recorded in MET-minutes per week (MET-min/wk) according to the IPAQ scoring protocol (www.ipaq.ki.se). IPAQ responses were converted into metabolic equivalents based on standard IPAQ scoring cutoffs and guidelines,[21] with METS values of 8.0, 4.0 and 3.3 min/wk corresponding to cut-offs for vigorous-intensity, moderate-intensity, and walking activities., respectively.

IL-6 was measured from blood plasma. Participants were fasting and free of strenuous exercise for a minimum of 24 hours prior to sampling and sat quietly for 10 minutes prior to having their blood drawn into Ethylenediaminetetraacetic acid tubes. Single 20 ml blood samples were collected between 10am and 2pm. Plasma was harvested within 1h of sampling and stored at −80°C until analysis. Levels of IL-6 were determined in duplicate using a high-sensitivity enzyme-linked immunosorbent assay (Quantikine HS; R & D Systems, Minneapolis, MN) that has a mean minimum detectable dose range of 0.016–0.011 pg/ml. Randomly selected samples were assayed in duplicate to monitor within plate and plate-to-plate variability. Coefficients of variations were less than 10%.

Three body composition measures including percent body fat, fat mass and lean body mass, were obtained by dual energy x-ray absorptiometry (GE Lunar DXA/GE Lunar Body Composition Software) and bioelectrical impedance (Bioimpedance Analyzer BIA model 450, Biodynamics Corporation Seattle WA). Height (cm) and body weight (kg) were measured to the nearest 0.25 cm and nearest 0.1 kg, respectively, using a floor model physician's scale/stadiometer.

### Statistical Analysis

Subject characteristics were described in terms of demographic and physiological variables including age, ethnicity, body composition variables (BMI, percent body fat, fat mass and lean body mass), metabolic equivalent tasks (MET's) and IL-6 levels (Table 1).

**Table 1:** Descriptive Statistics (n = 72).

	Sedentary Males (n = 12)	Male Recreational Athletes (n = 16)^1^	Serious Leisure Male Athletes (n = 44)^1^	Total Mean (Std. Dev.) or % (n = 72)
Mets**	311.7 (208.6)	774.4 (478.9)	4,466.9 (2,360.9)	2,954 (2,665)
Age*	28.9 (7.3)	34.4 (10.1)	38.5 (13.0)	36.0 (12.0)
%Body Fat	18.7 (5.6)	18.8 (8.1)	17.0 (6.6)	17.7 (6.6)
Fat Mass¥	15,875 (6,452)	16,767 (11,787)	13,820 (7,546)	14,783 (8,318)
Lean Mass#*	59,025 (6,316)	66,425 (9,845)	60,866 (7,282)	61,806 (8,042)
IL-6pg/ml**	1.33 (0.62)	0.95 (0.42)	0.69 (0.50)	0.86 (0.55)
Race				
African American	41.7% (5)	12.5% (2)	6.8% (3)	13.9% (10)
Caucasian	41.7% (5)	31.3% (5)	68.2% (30)	55.6% (40)
Asian	8.3% (1)	25.0% (4)	9.1% (4)	12.5% (9)
Hispanic	8.3% (1)	31.3% (5)	13.6% (6)	16.7% (12)
Other	0% (0)	0% (0)	2.3% (1)	1.4% (1)

Mean and SEM for all continuous variables and distributions of race, with descriptions for the entire sample and for groups separated by self-reported activity level (sedentary, recreational, serious leisure athletes). Statistically significant group differences are indicatied.

1 missing data varied by characteristic (0 or 1 for Male Recreational Athletes; 0–3 for Serious Leisure Male Athletes); no missing for Sedentary Males.

** p<.01, *p<.05 from comparison of groups by likelihood ratio chi square (for race) and ANOVA for other characteristics.

¥ Total body fat in grams.

# Total lean body mass in grams.

For further sample description, characteristics are also given for each of the three subsets of subjects by athletic activity levels. These subsets were compared using chi square or ANOVA as appropriate to distributional characteristics. In addition, as a basis for the structural modeling, Pearson correlation coefficients were computed between pairs of subject variables (Table 2).

**Table 2:** Correlations between Observed Variables (n = 72).

	Age	%BodyFat	FatMass gm	LeanMass gm	METs	IL-6 pg/ml
Age	1.00					
%BodyFat	0.50*	1.00				
FatMass gm	0.04	0.32*	1.00			
LeanMass gm	0.09	0.20	0.30*	1.00		
METs	0.16	−0.16	−0.20	−0.11	1.00	
IL-6 pg/ml	0.23*	0.38*	0.15	0.19	−0.35*	1.00

* p<.05.

Body fat positively correlated with age, fat mass and IL-6; Fat mass and lean mass positively correlated; Mets and IL-6 negatively correlated.

### Structural Equation Modeling

For analysis purposes, three variables were rescaled to provide greater consistency: METS values were divided by 10 and fat mass and lean mass values were divided by 100. The expectation –maximization (EM) algorithm was used to impute values for the nine instances of person-by-variable missing values (out of a total of 438 values in the data set) [22]. Univariate skewness and kurtosis were within acceptable limits (<2 for skewness and <7 for kurtosis). Preliminary analysis identified one extreme multivariate outlier; when this case was omitted, the Mardia multivariate normality index decreased substantially to 7.1, below the value (8) generally considered an acceptable level, considering that the maximum likelihood (ML) approach used in subsequent analyses is relatively robust with respect to moderate non-normality. [23]; [24] (Note also that we present robust model fit statistics.).

Structural equation modeling (SEM) with maximum likelihood (ML) estimation conducted with EQS software was used to estimate direct and indirect effects on IL-6 [25]. SEM allows examination of complex phenomena and can include estimation of both direct and indirect effects, as well as allowing multiple indicators of important constructs thus reducing the impact of measurement error [26]. SEM gives overall model fit statistics, thus controlling for inflation of Type I error due to multiplicity of tests from e.g. multiple applications of regression analysis [27]. Several model fit statistics were assessed, including: model chi square, comparative fit index (CFI), Bentler-Bonett non-normed fit index, and root mean-square error of approximation (RMSEA), as well as robust versions of these statistics and the Yuan-Bentler residual-based chi-square [28]; [29]. For RMSEA, a value closer to zero is desired; and for the others, higher values (closer to 1.0) indicate better fit. While there are no absolute cutoff criteria values associated with good fit, guidelines typically suggest that values greater than .90 or .95 are desired for concluding acceptable to good fit; and RMSEA values smaller than about .04 or .08–.10 indicate good and adequate fit, respectively, with the less strict end of the ranges acceptable in smaller samples [30]; [31].

Initially, a more saturated model was estimated which included all possible paths among age, METS, body composition, and IL-6. The two non-significant direct paths (from age to METS and age to IL-6) were omitted and the more parsimonious model was re-estimated, leading to a desired decrease in the model AIC [28]; [32]. The results from the parsimonious model are presented here.

This analysis was a secondary analysis of existing data (N = 72 in the analysis). The sample size was sufficient to detect medium effect sizes with power = .80 and alpha = .05, in terms of correlations (0.32) or in terms of specific relationships within the structural model. For SEM, the relatively small sample allowed rejection of poorly fitting models; however, as indicated above, robust versions of fit statistics and a parsimonious model were used.

## Results

### Description

A majority (56%) of the sample was Caucasian, with representation of other major racial/ethnic groups (Table 1). The average age was 36 years; the subset of serious leisure male athletes were somewhat older (average 38.5) than the subset of recreational athletes (34.4) and sedentary males (28.9). Body composition measures were typical of this age group, with sample averages of 18% body fat, 14,783 grams of fat mass, and 61,806 grams of lean mass. Average IL-6 level was 0.86 pg/ml. As would be expected, these parameters differed across the activity level subsets with serious leisure athletes with substantially higher levels of METs (4,467) compared to the other subsets (774 and 312).

Significant correlations were observed among body composition indicators (fat mass to percent body fat to fat mass at 0.32 and to lean mass at 0.30). Age was related to percent body fat (0.50) and to IL-6 (0.23) and IL-6 to METs (−0.35).

### Structural Equation Model

Significant correlations were observed among body composition indicators (fat mass to percent body fat to fat mass at 0.32 and to lean mass at 0.30). Age was related to percent body fat (0.50) and to IL-6 (0.23) and IL-6 to METs (−0.35).

**Figure 1:**
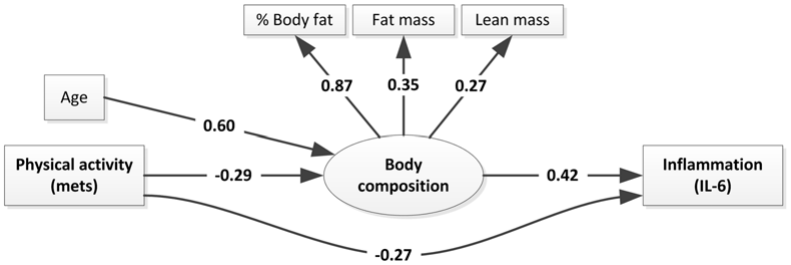
SEM Model of Physical Activity and age influences on inflammation via body composition;* indicates significant relationships (P<0.05).

The model in Figure 1 produced an acceptable fit with a desired non-significant model chi-square of 10.98 (df = 8, p = .20), CFI  = .94 (e.g. [30]), Bollen's IFI  = .95, and RMSEA of .07. Robust statistics also showed acceptable fit: Yuan-Bentler statistic = 9.87 (p = .27, non-significant p desired), CFI  = .90, Bollen's IFI = .91, and RMSEA of .08.

Considering the measurement portion of the model representing Body Composition from the three indicators, we see standardized coefficients showing weights of .87, .35, and .27 for indicators of body composition (%BodyFat, FatMass/100, and LeanMass/100, respectively). While the coefficient for lean mass was somewhat low for a factor loading, it was nevertheless statistically significant in the SEM. Note that as a sensitivity analysis, we did estimate an alternative model omitting LeanMass/100 as an indicator of body composition, but this produced no improvement in model fit.

Results indicate significant direct effects of METS and body composition on IL-6, with standardized coefficients of −.27 (p = .008) and .42 (p = .004), respectively. METS also has a direct effect on body composition (beta = −.29, p = .008) as does age (.60, p<.001). METS has an indirect effect on IL-6 through body composition (−.12, p = .049), and age also has an indirect effect on IL-6 through body composition (.25, p = .006).

## Discussion

This study demonstrated the influence of physical activity via body composition on IL-6 in a group of healthy males who ranged in activity level from athletic to sedentary. Age also showed an indirect effect on IL-6 via body composition, even though no direct effect of age on physical activity or IL-6 was found. Additionally, a direct effect of physical activity on IL-6 suggests the presence of pathways other than via body composition. The SEM approach demonstrated that previously-shown influences of physical activity on IL-6 likely operate through a combination of mechanisms, including body composition, and others which remain to be elucidated.

Findings of significantly lower inflammatory cytokine values with the more active subjects are consistent with previous studies of intensive physical activity [33]. Although all study participants were free of any known co-morbid conditions, evidence suggests that long-term exercise training reduces plasma inflammatory states [34], and vigorous levels of physical activity are associated with reduced levels of peripheral inflammatory mediators compared with a more sedentary lifestyle [35]. Exercise intervention programs reduce systemic low-level inflammation in patients with CVD and in healthy controls, but the studies did not investigate the relationship of body composition to this phenomenon [36].

The findings are unlikely to be influenced by acute changes in IL-6 following exercise, as the measurements were in a resting state with no exercise in the prior 12 hours. Acute moderate and strenuous intensity [37] exercise is associated with acute phase responses, with prolonged effects decreasing after exercise, suggesting cytokine inhibitors and anti-inflammatory cytokines may restrict the degree and extent of the inflammatory response to exercise. Following acute exercise, systemic levels of IL-6 demonstrate significant increases; however the precise change remains speculative with sex, exercise intensity [38], and age-based [39] differences.

The body composition pathway is consistent with the fact that adipose tissue directly influences IL-6 values. Adiposity is known to be an inflammatory condition with the percentage of IL-6 closely related to the pattern and degree of adiposity [40]. In healthy adults, the percentage of weight from body fat ranges from 6–30%, with women generally having more body fat than men [41]. In the present sample, the range of percentage of body fat was similar, with the athletic group demonstrating significantly lower IL-6 values, and the sedentary group in the normal range [42].

Changes in body composition from physical activity will likely alter muscle physiology in addition to adipose tissue. Skeletal muscles express cytokines that have direct autocrine and paracrine effects. IL-6 increases muscle protein degradation and is associated with lower muscle mass or strength and mobility disability and levels contribute to impaired mobility and functional decline in older persons [43]. Despite the influence of lean mass and adipose tissue on IL-6, this study demonstrates that after controlling for lean mass and body fat, physical activity remained strongly associated with differences in IL-6, which is potentially via change in muscle composition.

Alternative influences of body composition and exercise on inflammatory patterns may also be present. A recent prospective study assessing the direct effect of exercise training failed to show the improvement in the levels of IL-6 when weight loss was not observed [44], suggesting that the relationship shown in the present study between exercise and inflammatory profiles is not simple. Thus, while the current findings show relationships between variables at one point in time, the model will need to be fully tested with interventions that modify one or more of the independent variables, with regards to improving IL-6 measures.

### Limitations

Other factors not accounted for in this research design may account for individual differences in IL-6 including race and socio-economic status although this study was not specifically designed to examine these factors. Additionally, genetic factors been reported as important determinants for the individual response to anti-inflammatory effects of exercise training [45]. IL-6 was measured at only 1 point in time, and it is unclear whether these 1-time measurements resulted in valid estimates of subjects' longer term inflammatory status. Lastly, our study was based only in males, so it is unclear whether our results are generalizable to females.

The SEM model has been estimated from a small sample, at the lower limit (5 subjects per parameter) of recommendations (e.g Bentler & Chou [25]; [46]); nevertheless, the model achieved acceptable fit with statistically significant coefficients for direct and indirect paths of interest. Sensitivity analyses supported the stability of results: with the outlier left in the analysis or using only complete cases rather than imputation for missing values, coefficients were similar to those presented.

### Conclusion

We have shown that physical activity likely reduces inflammation [47] through improving body composition and through other, unknown pathways in healthy males. Age also influences inflammatory state through body composition. The active subjects were serious leisure athletes' including cyclists and triathletes, so these forms of physical activity appear to be beneficial with regards reducing inflammatory state. Our findings warrant further investigation and suggest that despite well known positive effects associated with physical activity, further mechanisms involving physical activity and reduced inflammation remain to be discovered. However, the present findings add to the evidence demonstrating the benefit of physical activity with regards influencing pathways that modify risk factors associated with inflammatory disorders.
